# Long-term body composition improvement in post-menopausal women following bariatric surgery: a cross-sectional and case–control study

**DOI:** 10.1530/EJE-21-0895

**Published:** 2021-12-08

**Authors:** Sara Santini, Nathalie Vionnet, Jérôme Pasquier, Michel Suter, Didier Hans, Elena Gonzalez-Rodriguez, Nelly Pitteloud, Lucie Favre

**Affiliations:** 1 Division of Endocrinology, Diabetology, and Metabolism, Lausanne University Hospital, Lausanne, Switzerland; 2 Center for Primary Care and Public Health (Unisanté), University of Lausanne, Lausanne, Switzerland; 3 Department of Visceral Surgery, Lausanne University Hospital, Lausanne, Switzerland; 4 Department of Surgery, Riviera-Chablais Hospital, Rennaz, Switzerland; 5 Faculty of Biology and medicine, University of Lausanne, Lausanne, Switzerland; 6 Interdisciplinary Center for Bone Diseases, Lausanne University Hospital, Lausanne, Switzerland

## Abstract

**Objective:**

Bariatric surgery (BS) induces loss of body fat mass (FM) with an inexorable loss of lean mass (LM). Menopause leads to deleterious changes in body composition (BC) related to estrogen deficiency including LM loss and increase in total and visceral adipose tissue (VAT). This study aims to describe the long-term weight evolution of post-menopausal women after Roux-en-Y gastric bypass (RYGB) and to compare the BC between BS patients vs post-menopausal non-operated women.

**Design:**

Cross-sectional study of 60 post-menopausal women who underwent RYGB ≥2 years prior to the study with nested case–control design.

**Methods:**

Post-menopausal BS women were matched for age and BMI with controls. Both groups underwent DXA scan, lipids and glucose metabolism markers assessment.

**Results:**

Median follow-up was 7.5 (2–18) years. Percentage of total weight loss (TWL%) was 28.5 ± 10%. After RYGB, LM percentage of body weight (LM%) was positively associated with TWL% and negatively associated with nadir weight. Forty-one post-BS women were age- and BMI-matched with controls. Post-BS patients showed higher LM% (57.7% (±8%) vs 52.5% (±5%), *P*  = 0.001), reduced FM% (39.4% (±8.4%) vs 45.9% (±5.4%), *P*  < 0.01) and lower VAT (750.6 g (±496) vs 1295.3 g (±688), *P*  < 0.01) with no difference in absolute LM compared to controls. While post-BS women showed a better lipid profile compared to controls, no difference was found in glucose markers.

**Conclusions:**

Post-menopausal women after RYGB have a lower FM and VAT, preserved LM and a better lipid profile compared to controls. Weight loss after RYGB seems to have a persistent positive impact on metabolic health.

## Introduction

Obesity is associated with numerous health conditions including cardiovascular disease, diabetes, and several cancers (1). The dramatic worldwide increase of the prevalence of obesity during the last decades has become a major public health problem (2). While lifestyle intervention alone generally fails to achieve sustained long-term weight loss, bariatric surgery (BS) has proven to be an effective therapy for sustained weight loss in severe obesity (3, 4). Roux-en-Y gastric bypass (RYGB) is one of the most commonly performed surgeries worldwide. It is a restrictive and malabsorptive surgery that results in a decrease in body fat mass (FM) including visceral adipose tissue (VAT) and an improvement in metabolic parameters and cardiovascular risk (5). However, it also leads to inexorable loss in lean mass (LM), ranging from 10 to 45% 12 months after RYBG (6, 7, 8, 9, 10, 11, 12, 13, 14, 15). While long-term studies on body composition (BC) after BS are scarce, significant loss in LM during the first 18 months post-RYGB and a tendency for LM stabilization or slight increase afterwards have been reported (6, 15, 16). Loss in LM may have deleterious metabolic consequences. Indeed, LM contributes to insulin-mediated glucose uptake (17), increases resting energy expenditure and high-density lipoprotein cholesterol levels and produces myokines that prevent metabolic deterioration (17, 18). LM is also essential for muscular strength and the body’s functional capacity (19). Thus, counseling for dietary protein and micronutrients intake as well as regular physical activity is part of the long-term treatment for surgical patients in order to limit LM loss (20, 21). Studies comparing BC in RYGB-operated patients vs age- and BMI-matched controls are limited, reporting similar (22, 23) or higher LM in the surgical group (24, 25). So far, no guidelines are available to define the pathological loss of LM during follow-up. Female sex, age and malnutrition are generally considered as risk factors for LM loss (26).

Menopause is characterized by the lack of menstrual cycles for more than a year and occurs at an average age of 50 years (27). It is accompanied by a significant increase in body weight related to estrogen deficiency. In particular, menopause is associated with an increase in FM and a loss of LM (28, 29). The stronger increase in VAT after menopause leads to a shift from a gynoid to an android pattern of fat distribution (28). Recent study indicates that hormone replacement therapy (HRT) improves BC in post-menopausal women (29).

In this study, we aimed first to describe the long-term body weight and metabolic evolution of a post-menopausal cohort with previous RYGB, and secondly, to compare the BC between two post-menopausal populations: bariatric women vs non-operated controls, matched for age and BMI. Herein, we show that post-menopausal women, at least 2 years after RYGB, have a decreased FM% and VAT, conserved LM% and a better lipid profile as compared to controls.

## Methods

### Study design and participants

This is a cross-sectional descriptive cohort with a nested case–control design. The study was performed in a single academic center, at the Centre Hospitalier Universitaire Vaudois (CHUV) in Lausanne.

BS patients were recruited from the Cohort Obesity of Lausanne (COOL) which is a longitudinal observational cohort established from 2015 for all patients operated at the CHUV since 1999 to investigate long-term effects of BS (30). All bariatric procedures were performed after a complete and multidisciplinary evaluation at CHUV and eligible patients presented with a BMI ≥40 or ≥35 kg/m^2^ with the presence of at least one comorbidity and failure of conservative treatment for over 2 years (31). Laparoscopic RYGB was performed by the same surgical team by creating a 15 mL gastric pouch, a retrocolic 100–150 cm Roux alimentary limb, a 21-mm circular stapled gastrojejunostomy and a linear stapled jejunostomy. All patients were advised on diet and physical activity and received supplements (daily multivitamin and calcium 1000 mg/vitamin D 800 UI) according to Swiss guidelines on obesity (31). Inclusion criteria were women aged >50 years with one BC analysis by dual-energy X-ray absorptiometry (DXA) at least 2 years post-surgery and persistent amenorrhea for >12 months at the time of the DXA. Finally, as it has previously been reported that nadir weight after RYGB is usually reached between the first and the second year after surgery (3), patients with a follow-up shorter than 2 years after surgery were excluded. Patients with established alcohol dependence after surgery were excluded, possibly impacting the LM. Sixty post-menopausal women were eligible out of 396 (Supplementary Fig. 1, see section on supplementary materials given at the end of this article).

Non-operated controls were selected from OsteoLaus, another local cohort of 1475 post-menopausal women 50 to 80 years, enrolled from general population, which aims to study bone health (32). These controls were enrolled as they had a BC analysis performed in the same DXA device as in the COOL cohort (*n* = 1234). OsteoLaus data were collected between March 2015 and February 2018.

All participants (operated and controls) were post-menopausal at the time of the DXA and were questioned on current menopausal HRT use. OsteoLaus and COOL studies were approved by the Institutional Ethics Committee of the University of Lausanne, and written informed consent was obtained from all participants.

### Study variables

Anthropometric measurements and medical history were collected at the time of the DXA examination for all participants. In operated patients, data were collected before RYGB as well. Participants were weighed barefoot in light clothes (0.2–0.4 kg) with a precision of 0.1 kg. Height was measured with a fixed wall stapediometer with a precision of 0.1 cm. Excess weight was defined as the pre-surgical weight minus ideal body weight (based on a BMI of 25 kg/m^2^). The % excess weight loss (EWL%) was defined as the pre-surgical weight minus the follow-up weight, divided by excess weight and multiplied by 100. The % total weight loss (TWL%) was defined as the pre-surgical weight minus the follow-up weight divided by the pre-surgical weight and multiplied by 100. Nadir weight was defined as the lowest weight achieved during follow-up.

Weight regain was calculated as the post-operative weight minus the nadir weight divided by the nadir weight and multiplied by 100. Dyslipidemia was defined as the presence of one or more abnormal serum lipid concentrations (LDL >3 mmol/L, triglycerides >2.3 mmol/L) or use of statins (33). The diagnosis of diabetes (type 2 diabetes (T2D)) included glycated haemoglobin (HbA1c) ≥6.5%, fasting glycemia ≥7 mmol/L, a 2-hour glycemia during 75 g oral glucose tolerance test ≥11.1 mmol/L or the use of diabetes medication. T2D remission was defined as an HbA1c level <6.5% and a fasting glucose concentration <7 mmol/L in the absence of any antidiabetic medication.

Standard biological assays were performed in the accredited clinical chemistry laboratory of CHUV. Glucose, HbA1c, insulin, hepatic tests (aspartate transaminase, alanine transaminase, gamma-glutamyl transferase), lipids, thyroid-stimulating hormone, uric acid, albumin and creatinine were measured after an overnight fast in a range of 6 months before or after DXA in both cohorts. Blood tests of controls were used for comparison when available.

BC analysis was assessed in both groups using Lunar iDXA (GE Healthcare) (7). Participants were placed centered on the scanning field in a supine position, with palms down and arms at sides, slightly separated from the trunk. Regions of interest included total body, trunk, android, gynoid, upper limbs and lower limbs. The android region lower boundary was defined at the top of the iliac crest and the upper boundary as 20% of the distance between the pelvis and neck cuts. The upper boundary of the gynoid region was set below the pelvis cut at 1.5× android height. Gynoid height is determined as 2× android region height (34). For each region, total mass, FM and LM were calculated by the Lunar iDXA software. VAT was determined using DXA CoreScan software which has been demonstrated to be highly reliable compared to MRI (35). For the study, total FM and LM, android and gynoid FM and LM and VAT were evaluated. FM and LM were expressed as absolute values and as a percentage of body weight. Three indexes were calculated: appendicular lean mass index (ALMI) was calculated as the ratio of the addition of the four limbs LM (kg) over height (m) squared; lean mass index (LMI) was calculated by dividing LM (kg) by the square of height (m); fat mass index (FMI) was calculated by dividing total FM (kg) by the height (m) squared.

Different definitions have been published for sarcopenia diagnosis, which is consensually defined by a loss of muscle mass and muscle strength. Cut-off values for defining sarcopenia according to ALMI vary between 4.42 and 5.67 kg/m^2^ in women (36, 37). We used the values below 5.67 kg/m^2^ to exclude sarcopenia, as previously shown (37).

### Statistical analysis

Standard descriptive analyses were used to summarize the study variables (e.g. frequencies and percentages for categorical variables and means ± s.d. for continuous variables).

In order to find the predictors of LM percentage (LM%) and ALMI in the post-RYGB cohort, we examined age, time of follow-up, TWL% and nadir weight as predictor variables using univariable and multivariable linear regressions. In the multivariable model, the variables were centered and we considered an interaction factor between TWL% and nadir weight in addition to the four predictor variables. VAT was compared between participants with and without T2D before RYGB using the Student’s *t*-test. The same comparison was made by adjusting for FM percentage of total body mass (FM%) using multivariable linear regression. The same analyses were conducted between patients with and without T2D after RYGB.

The patients who underwent RYGB were matched for age and BMI at the time of their BC analysis with non-operated controls at a ratio of 1:1 using the propensity scores. The propensity scores were estimated using a logistic regression. Optimal pair matching was performed using the MatchIt package (38) in the R statistical software. Participants outside the common support region were discarded in analyses comparing the two groups. Continuous and categorical variables were compared between the two groups using Student's *t*-test and Fisher's exact test, respectively. For continuous variables, adjusted differences for age and BMI were calculated using linear regression.

## Results

### Description of long-term body weight evolution in the post-menopausal RYBG cohort

In the complete bariatric group (*n* = 60), mean age was 57 (±5.7) years, median follow-up was 7.5 (2–18) years after RYGB, and mean age at the time of surgery was 48.8 (±6.8) years. The TWL% was 28.48 (±9.97)% and the mean EWL% was 67.46 (±29.24)%. The nadir weight was reached after a mean of 24 (5–162) months. Prevalence of metabolic comorbidities in patients before and after RYGB at follow-up time is shown in Table 1. Remission rate after RYGB for T2D, hypertension and dyslipidemia were 68.7 (*n* = 11/16), 61.8 (*n* =21/34) and 68.0% (*n* =34/50), respectively.

**Table 1:** Weight evolution of operated patients. Data expressed as mean (s.d.) or median (min–max). TWL, total weight loss defined as the operative weight minus the follow-up weight divided by the operative weight and multiplied by 100. EWL, excess weight loss defined as the operative weight minus the follow-up weight, divided by excess weight and multiplied by 100. Nadir weight defined as the lowest weight achieved during the follow-up.

Variable	Before RYGB (*n* = 60)	Post RYGB (*n* = 60)
Weight, (kg)	117.27 (22.17)	83.9 (20)
BMI, (kg/m^2^)	45.92 (7.29)	32.9 (7.4)
Follow-up, (years)	–	7.5 (2–18)
Weight loss, (kg)	–	33.4 (14.3)
TWL, (%)		28.5 (10)
EWL, (%)	–	67.4 (29.2)
Nadir weight, (kg)	–	73.4 (13.7)
Time of nadir weight, months	–	24 (5–62)
Type 2 diabetes, *n* (%)	16 (26.7)	6 (10.0)
Hypertension,* n* (%)	34 (56.7)	14 (23.3)
Dyslipidemia, *n* (%)	50 (83.3)	16 (26.7)

**Figure 1:**
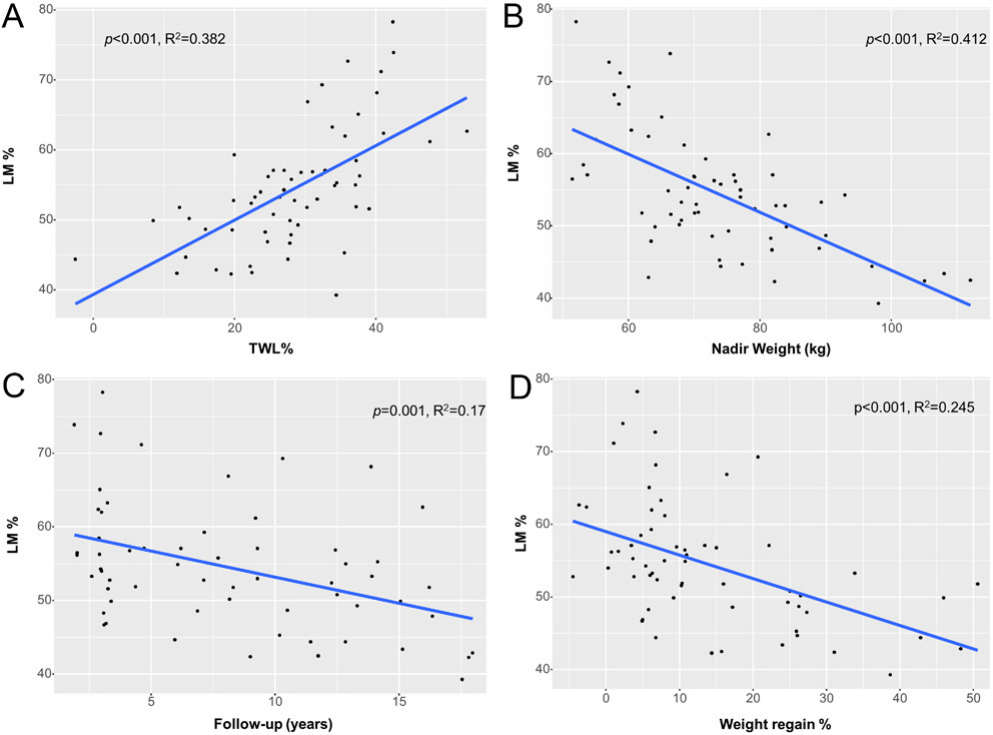
Univariable linear regressions of the association between percentage of lean mass on total body mass (LM%) as outcome variable, and % total weight loss (TWL%) (A), nadir weight (B) time of follow-up (C) and weight regain (D) as predictor variables in the RYGB cohort. R^2^, Spearman’s coefficient. A full color version of this figure is available at https://doi.org/10.1530/EJE-21-0895.

**Figure 2:**
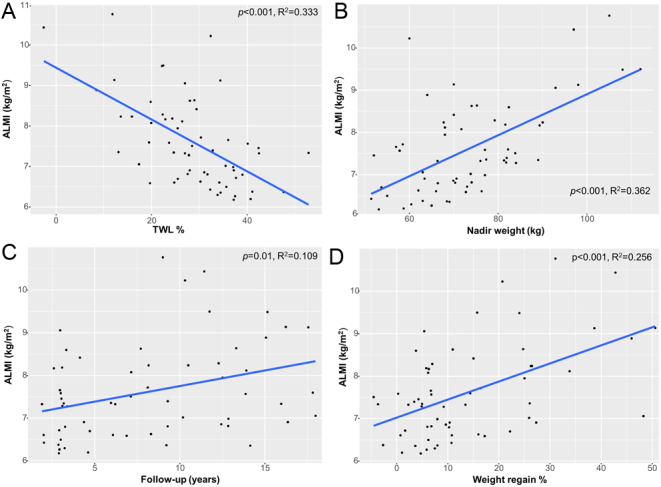
Univariable linear regressions of the association between appendicular lean mass index (ALMI) as outcome variable and % total weight loss (TWL%) (A), nadir weight (B), time of follow-up (C) and weight regain (D) as predictor variables, in the RYGB cohort. R^2^, Spearman’s coefficient. A full color version of this figure is available at https://doi.org/10.1530/EJE-21-0895.

After RYGB, LM% was positively associated with TWL% and negatively associated with nadir weight, time of follow-up and weight regain using univariable regression (Fig. 1). As LM% was associated with the time of follow-up and possibly influencing body weight evolution, a multiple linear regression model, adjusted for age and time of follow-up was used, showing that LM% was positively correlated with TWL% and negatively associated with nadir weight (Supplementary Table 1). Conversely, ALMI was negatively associated with TWL% and positively associated with nadir weight both in univariable and multiple linear regression analysis (Fig. 2 and Supplementary Table 2). ALMI was also directly associated with the extent of weight regain.

Concerning the relationship between VAT and metabolic comorbidities, women with T2D before surgery (*n* = 16) showed a significantly higher VAT after RYGB compared to operated patients without T2D before surgery (1473 ± 1130 g vs 902 ± 677 g,* P* = 0.02). Similarly, women with T2D after surgery (*n* = 6) showed a greater amount of VAT compared to non-diabetic women (1997 ± 1667 g vs 949 ± 657 g, *P*  < 0.01). These data were confirmed by multivariable analysis after adjusting for FM% (*P*  = 0.02 and *P*  < 0.01 for T2D before and after surgery, respectively). No significant difference in VAT was found according to the presence or absence of hypertension and dyslipidemia before and after RYGB (data not shown).

### Improved body composition in post-menopausal RYGB women versus age and BMI-matched controls

As the operated patients had a higher BMI and younger age than the controls, we were able to match on an individual basis a subsample of 41 out of the 60 patients from the surgical group to 41 controls (Supplementary Fig. 1). Mean age was 58.4 (±6.2) years vs 59.4 (±3.2) years (*P*  = 0.4) and mean BMI was 29.6 (±4.9) kg/m^2^ vs 31.1 (±5.6) kg/m^2^ (*P*  = 0.2) in operated patients vs controls, respectively. Two out of 41 post-RYGB women were on HRT vs 8/41 in controls, *P*  < 0.04 (Table 2).

**Table 2:** Body composition results of operated patients (post-RYGB) vs controls. Data are expressed as mean (s.d). Comparisons were adjusted for BMI and age (adjusted* P*-value).

Variable	Post-RYGB (*n* = 41)	Controls (*n* = 41)	Raw difference	*P* -value	Adjusted difference	Adjusted *P* -value
Age, (years)	58.4 (6.2)	59.4 (3.2)	1 (1.1)	NA	0.47 (1.0)	NA
BMI, (kg/m^2^m)	29.63 (4.99)	31.08 (5.65)	1.45 (1.2)	NA	1.02 (1.1)	NA
HRT, *n* (%)	2/41 (4.9%)	8/41 (19.5%)	**–**	**0.04**	–	–
Total body mass, (kg)	74.7 (11.8)	80.6 (14.5)	5.9 (2.9)	**0.05**	2.8 (1.4)	**0.05**
Total FM, (kg)	30.9 (9.9)	36.52 (10.3)	6.4 (2.2)	**0.005**	3.9 (0.9)	**<0.001**
Total FM, (%)	39.4 (8.4)	45.88 (5.3)	6.46 (1.6)	**<0.001**	4.89 (0.9)	**<0.001**
Total LM, (kg)	42.4 (4.7)	41.7 (4.8)	−0.7 (1.0)	0.5	−1.3 (0.9)	0.18
Total LM, (%)	57.7 (8.0)	52.5 (5.0)	− 5.14 (1.5)	**<0.001**	−3.7 (0.9)	**<0.001**
Trunk FM, (kg)	14.5 (6.0)	19.7 (6.4)	5.2 (1.3)	**<0.001**	3.7 (0.5)	**<0.001**
Trunk FM, (%)	39.4 (11.2)	48.4 (7.6)	−7.3 (2.3)	**<0.001**	−5.9 (1.3)	**<0.001**
Trunk LM, (kg)	20.7 (2.4)	19.5 (2.2)	−1.2 (0.5)	**0.02**	− 1.4 (0.5)	**0.005**
Trunk LM, (%)	58.8 (10.9)	51.6 (7.6)	−7.3 (2.2)	**0.002**	−5.9 (1.3)	**<0.001**
Android FM, (kg)	2.4 (1.2)	3.5 (1.3)	1.1 (0.3)	**<0.001**	0.8 (0.1)	**<0.001**
Android FM, (%)	40.8 (12.7)	51.5 (9.0)	10.8 (2.4)	**<0.001**	8.3 (1.5)	**<0.001**
Android LM, (kg)	3.2 (0.4)	3.1 (0.4)	−0.1 (0.9)	0.1	−0.2 (0.9)	**0.04**
Gynoid FM, (kg)	5.0 (1.5)	6.1 (1.6)	1.1 (0.4)	**0.002**	0.7 (0.2)	**<0.001**
Gynoid FM, (%)	44.6 (7.5)	48.2 (4.9)	3.6 (1.4)	**0.01**	2.4 (1.1)	**0.03**
Gynoid LM, (kg)	6.1 (0.8)	6.4 (0.8)	0.3 (0.2)	0.06	0.3 (0.1)	0.1
FMI	11.7 (3.9)	14.0 (4.0)	2.3 (0.9)	**0.008**	1.3 (0.2)	**<0.001**
ALMI	7.2 (0.9)	7.4 (1.0)	0.2 (0.2)	0.4	−0.01 (0.1)	0.9
LMI	16.4 (1.6)	16.0 (1.7)	−0.4 (0.4)	0.26	−0.7 (0.3)	**0.005**
VAT, (g)	751 (496)	1 295 (688)	544 (132)	**<0.001**	423 (84)	**<0.001**

*P*-values <0.05 are displayed in bold.

ALMI, appendicular lean mass index appendicular (lean mass/squared height); FM, fat mass; FMI, fat mass index (fat mass/ squared height); HRT, hormone replacement therapy; LM, lean mass; LMI, lean mass index (lean mass/squared height); NA: not applicable; VAT, visceral adipose tissue.

**Figure 3:**
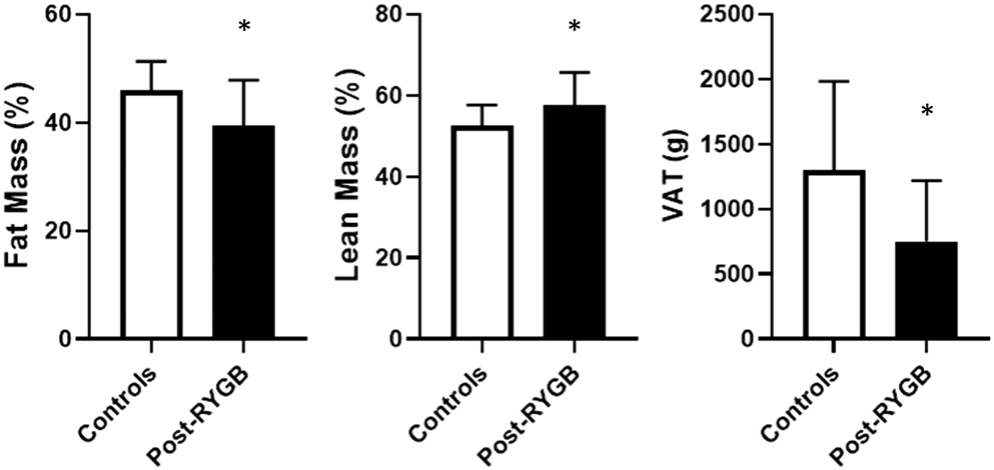
Differences in % fat mass of total body weight, % lean mass of total body mass and visceral adipose tissue (VAT) in post-RYGB women patients vs controls. Data are expressed as mean ± s.d. Statistical analysis was done using Student’s *t*-test. *P -value <0.001.

Post-RYGB women exhibited significantly decreased total FM% (39.4 ± 8.4% vs 45.9 ± 5.4%, *P*  < 0.01) associated with lower VAT (751 ± 496 g vs 1 295 ± 688 g, *P*  < 0.001) and android fat (40.8 ± 12.7% vs 51.5 ± 9%, *P*  < 0.001) as compared to non-operated controls (Fig. 3 and Table 2). Consequently, post-RYGB women had higher LM% (57.7 ± 8% vs 52.5 ± 5%, *P*  < 0.001). Except for the trunk region, there was no difference in any regional LM. Absolute LM, ALMI and LMI were similar in both groups and none of the post-RYGB women had pathological ALMI, thus ruling out the diagnosis of sarcopenia in this group. When comparisons were further adjusted for BMI and age, LMI and android LM were significantly higher in the operated group compared to the controls.

### Improved lipid profile in post-menopausal women after RYGB vs age and BMI-matched controls

Post-RYGB women showed a better lipid profile compared to controls (total cholesterol 4.8 ± 0.9 mmol/L vs 5.5 ± 0.9 mmol/L, *P*  < 0.001; LDL 2.4 ± 0.8 mmol/L vs 3.4 ± 0.8 mmol/L, *P*  < 0.001; HDL 1.9 ± 0.4 mmol/L vs 1.6±0.4 mmol/L *P*  = 0.008) with a reduced prevalence of dyslipidemia (*P*  = 0.02). No significant difference was found in the prevalence of T2D between the two groups (Table 3). Fasting glucose levels and Homeostatic Model Assessment for Insulin Resistance were similar between groups.

**Table 3:** Clinical parameters of post-RYGB patients and non-operated controls.

Variable	Post-RYGB (*n* = 41)	Controls (*n* = 41)	*P* -value
Glucose, (mmol/L)	5.2 (0.9)	5.4 (1.1)	0.3
Insulin, (mmol/L)	7.5 (4.5)	12.1 (10.6)	0.06
HOMA-IR	1.7 (1.1)	3.1 (3.6)	0.07
Uric acid, (µmol/L)	264.3 (64.8)	289.8 (62.7)	0.08
Total cholesterol, (mmol/L)	4.8 (0.9)	5.5 (0.9)	**<0.001**
LDL, (mmol/L)	2.4 (0.8)	3.4 (0.8)	**<0.001**
HDL, (mmol/L)	1.9 (0.4)	1.6 (0.4)	**0.008**
Triglycerides, (mmol/L)	1.2 (0.5)	1.2 (0.4)	0.1
TSH, (mU/L)	2.6 (1.2)	2.9 (1.3)	0.4
Albumin, (g/L)	42.8 (3.9)	42.5 (2.4)	0.7
Creatinine, (µmol/L)	65.1 (15.7)	70.1 (8.8)	0.08
AST, (U/L)	25.9 (7.4)	22.1 (5.9)	**0.02**
ALT, (U/L)	25.8 (9.5)	23.9 (9.2)	0.4
γGT, (U/L)	19.6 (10.5)	24.7 (22.2)	0.2
Type 2 diabetes, *n* (%)	3/39 (7.7)	3/39 (7.7)	1
Hypertension, *n* (%)	10/39 (25.6)	14/39 (35.9)	0.46
Dyslipidemia, *n* (%)	13/39 (33.3)	24/39 (61.5)	**0.02**

Data expressed as mean (s.d). *P* -values <0.05 are displayed in bold.

ALT, alanine transaminase; AST, aspartate transaminase; HDL, high density lipoprotein; HOMA-IR, homeostatic model assessment for insulin resistance; LDL, low density lipoprotein, TSH, thyroid-stimulating hormone; γGT, gamma-glutamyl transferase.

## Discussion

The results of the present study indicate a favorable long-term BC after RYGB in post-menopausal women. Indeed, we demonstrated decreased FM percentage and VAT content and conserved LM in operated women as compared to age- and BMI-matched controls. This is an important finding as the majority of the patients who undergo BS are women. To date, no other study has specifically investigated the changes in BC after BS in post-menopausal patients. However, two studies with slightly younger populations investigating the changes in BC after RYGB in both males and females also showed higher LM% and reduced FM% 2 years following surgery vs BMI-, age- and sex-matched controls (24, 25). Yet, the menopausal status was not reported. Other studies with similar design found no significant difference in BC between groups (22, 23, 39). Differing methodologies (DXA, bioimpedance and plethysmography) could account for the inconsistent results (22, 25); bioimpedance being affected by hydration and plethysmography leading to an underestimate of FM in obesity (40). In addition, the wide range of follow-up interval for RYBG from 12 months (23) to 2–5 years (22, 24, 25) could also influence the results, as also shown in the present study (Figs 1 and 2). The difference found in FM and VAT are partially consistent with a previous study, which reported similar results with VAT, but not with other body fat measurements (41).

This study demonstrated that absolute LM value, LMI and ALMI were not significantly different between operated vs non-operated women. BMI was not significantly different between the two groups. However, as the mean difference in BMI was 1.5 point and 1 year in age, all comparison in BC compartments were further adjusted for BMI and age. After adjustment, all results were confirmed and we found that LMI was significantly higher in post-RYGB patients.

Thus, it was important and unexpected to show the positive effects of RYGB on LM preservation in post-menopausal patients, as post-menopausal bariatric populations might present multiple risk factors for LM loss and sarcopenia including low estrogen, age, physical inactivity, low protein intake and hypovitaminosis D (42). Existing literature on the effect of HRT on BC is controversial, showing either absence or a beneficial effect on BC. Recent data from the large OsteoLaus cohort demonstrated that HRT was associated with reduced android and visceral FM with no benefit on LM (29). In the present study, despite a higher prevalence of HRT, the control group showed a greater amount of FM, android FM and VAT. Thus, we do not believe that HRT represents a confounding factor that could affect BC results.

As LM is inversely related to factors associated with cardiovascular risk (17) and VAT expansion increases cardiometabolic risk (43), we can postulate that improved BC after RYGB could protect these patients in the long-term against metabolic and cardiovascular complications (44). Moreover, LM is the main determinant of basal metabolism (19) and it contributes to long-term preservation of body weight. The beneficial effect of RYGB on metabolic parameters was also shown herein by the significant remission rates for T2D, hypertension and dyslipidemia after surgery in the operated group. We described a higher VAT in operated women with T2D at the time of surgery as compared to non-diabetic. Previous studies reported that diabetic patients have greater amount of VAT before surgery compared to normoglycemic subjects (7). These data highlight the association between fat distribution and T2D, even later after surgery, via the well-known lipolytic and pro-inflammatory profile of the VAT.

However, when we compared the surrogate markers of insulin resistance between controls and RYGB patients, we could not detect a significant difference, as previously shown (24, 41). The discrepancy between our results and previous reports may be due to the differences in sex and age, with a possible negative effect of estrogen deficit and aging on insulin sensitivity, mitigating glucose outcomes after RYGB in our cohort. Yet, we showed that operated patients displayed a better cholesterol profile. Our data are consistent with a previous study comparing 69 post-RYGB patients (BMI<30 kg/m^2^, 2 years after RYGB) with age-, sex- and BMI-matched controls demonstrating lower LDL and FM associated with higher LM (24).

The present study highlights several predictors of LM changes after RYGB. A regression analysis adjusted for age and time of follow-up demonstrated that a lower nadir weight was associated with greater LM%. Consistently, LM% was positively correlated with TWL%. This could be explained by an increased muscle mass possibly associated with increased physical activity. Alternatively, post-RYGB patients might preferentially lose FM rather than LM. This is consistent with results from Davidson *et al*. (6) showing that LM% of post-RYGB patients increased between 1 and 5 years after RYGB. Taken together, these data suggest a predictive value for nadir weight and TWL% on LM%, later after RYGB. LM% was also negatively influenced by time of follow-up, reflecting a possible loss of LM over time. In contrast to LM%, higher nadir weight and lower TWL% predicted greater ALMI, suggesting that this functional compartment of muscle mass could be differentially affected by weight loss after surgery, compared to full body LM. This hypothesis is supported by the higher amount of trunk LM found post-RYGB in operated patients compared to controls (Table 2). Further, Heshka *et al.* demonstrated that RYGB patients have greater trunk organ mass and lower skeletal mass compared to non-operated controls (39).

As expected, LM% negatively correlate with weight regain suggesting a subsequent redistribution in BC with an increase in FM percentage. However, weight regain predicted for a greater ALMI. This is consistent with an expansion in both LM and FM.

### Strengths and limitations

The strengths of this study are the use of high-quality gold-standard methods for BC, the analysis of a specific subpopulation, post-menopausal women, matched with controls and the long follow-up period after surgery. Limitations include a modest sample size in the subsample of 41 matched surgical patients to controls and the exclusion of surgical patients with higher BMI and/or younger age. In particular, the results on VAT analysis in the surgical group according to diabetes might be affected by extreme values and the small sample size. Another limitation consists in the lack of preoperative BC data with the analysis of the actual evolution after surgery. Patients and controls were not matched for ethnicity, as this information was not available. However, Heymsfield *et al.* (42) established that race is not a major contributor of BC proportions when BMI is held constant. Although all operated patients received the same nutrition and physical activity recommendation, we did not collect specific data on physical activity levels and food intake.

### Conclusion

RYGB in women lead to an improvement of BC that persists in the long-term. Preservation in LM after RYGB might indicate a low risk of sarcopenia in post-menopausal women. While these findings appear promising for BS patients, further studies are warranted to assess BC evolution in the long-term in elderly population.

## Supplementary Material

Supplementary Figure 1. Flow-chart describing participants’ inclusion.

Supplementary Table 1. Multiple linear regression adjusted for age and time of follow-up studying the association between percentage of total LM body mass (outcome variable) TWL% and nadir weight (predictor variables) in the RYGB cohort. TWL%, total weight loss.

Supplementary Table 2. Multiple linear regression studying the association between Appendicular Lean Mass Index, ALMI, (outcome variable) and age, time of follow-up, TWL% and nadir weight (predictor variables) in the RYGB cohort. TWL%, total weight loss.

## Declaration of interest

The authors declare that there is no conflict of interest that could be perceived as prejudicing the impartiality of this study.

## Funding

This work did not receive any specific grant from any funding agency in the public, commercial or not-for-profit sector.
