# Supplementary material for: Long-term body composition improvement in post-menopausal women following bariatric surgery: a cross-sectional and case–control study
Source: Eur J Endocrinol. 2021 Dec 8;186(2):255–63. doi: 10.1530/EJE-21-0895 (PMC8789027; doi:10.1530/EJE-21-0895)
Supplement: Supplementary Figure 1. Flow-chart describing participants’ inclusion. [file supplementary_figure_1.pdf]

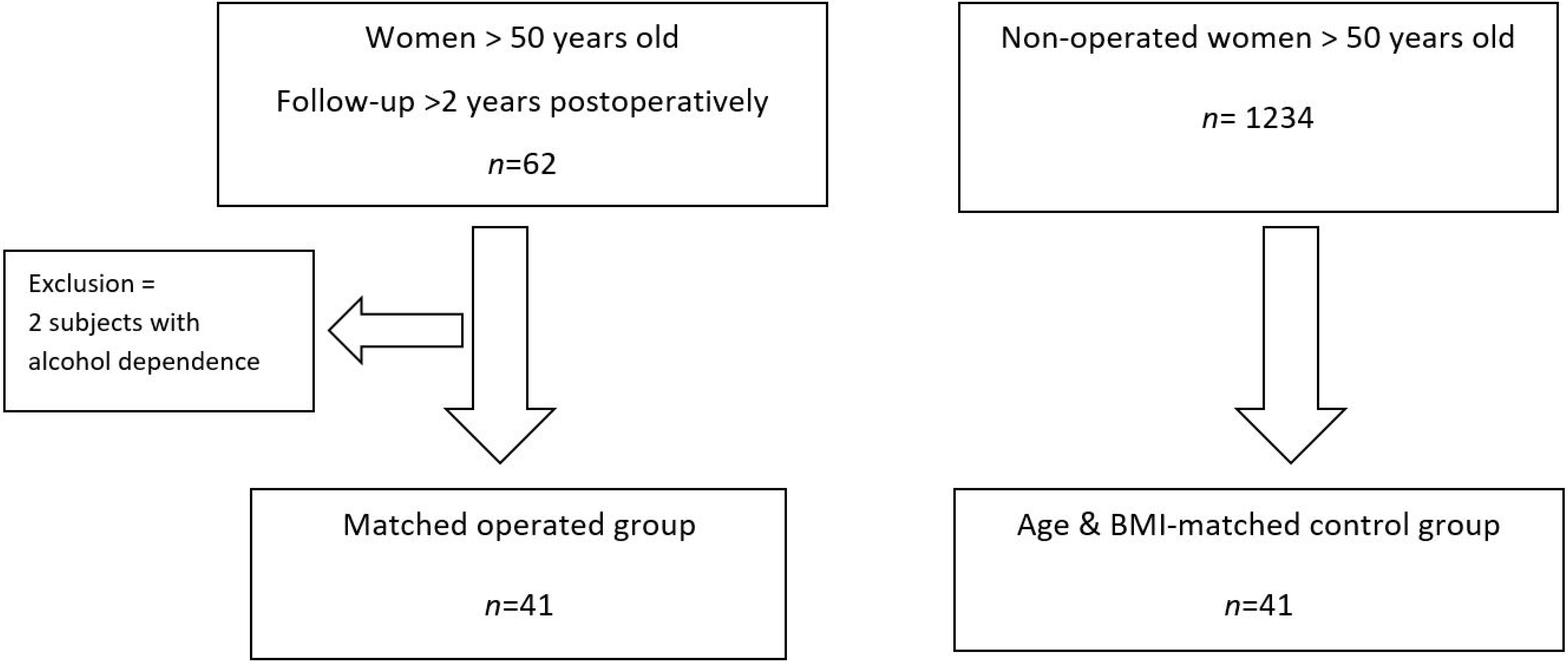
Women > 50 years old
Follow-up >2 years postoperatively
n=62
Non-operated women > 50 years old
n= 1234
Exclusion =
2 subjects with
alcohol dependence
Matched operated group
n=41
Age & BMI-matched control group
n=41
