# Supplementary material for: Long-term body composition improvement in post-menopausal women following bariatric surgery: a cross-sectional and case–control study
Source: Eur J Endocrinol. 2021 Dec 8;186(2):255–63. doi: 10.1530/EJE-21-0895 (PMC8789027; doi:10.1530/EJE-21-0895)
Supplement: Supplementary Table 1. Multiple linear regression adjusted for age and time of follow-up studying the association between percentage of total LM body mass (outcome variable) TWL% and nadir weight (predictor variables) in the RYGB cohort. TWL%, total weight loss. [file supplementary_table_1.pdf]

Term	Beta coefficient	95% Confidence interval	*p*-value
(Constant)	53.723	52.229 – 55.216	<.001
Total weight loss (TWL%)	0.369	0.211 – 0.526	<.001
Nadir weight	-0.305	-0.421 – -0.190	<.001
Follow-up (years)	-0.285	-0.585 – 0.015	0.062
Age (years)	0.009	-0.240 – 0.259	0.94
TWL%* Nadir weight	-0.014	-0.025 – -0.004	0.009

Supplementary Table 1. Multiple linear regression adjusted for age and time of follow-up studying the association between percentage of total LM body mass (outcome variable) TWL% and nadir weight (predictor variables) in the RYGB cohort. TWL%, total weight loss.
