# Supplementary material for: Long-term body composition improvement in post-menopausal women following bariatric surgery: a cross-sectional and case–control study
Source: Eur J Endocrinol. 2021 Dec 8;186(2):255–63. doi: 10.1530/EJE-21-0895 (PMC8789027; doi:10.1530/EJE-21-0895)
Supplement: Supplementary Table 2. Multiple linear regression studying the association between Appendicular Lean Mass Index, ALMI, (outcome variable) and age, time of follow-up, TWL% and nadir weight (predictor variables) in the RYGB cohort. TWL%, total weight loss. [file supplementary_table_2.pdf]

**Term**	**Beta coefficient**	**95% Confidence interval**	***p*-value**
(Constant)	7.535	7.314 – 7.755	<.001
Total weight loss (TWL%)	-0.037	-0.06 – -0.014	0.002
Nadir weight	0.03	0.013 – 0.047	<.001
Follow- up (years)	0.047	0.003 – 0.091	0.038
Age (years)	-0.009	-0.046 – 0.028	0.622
TWL%* Nadir weight	-0.001	-0.003 – 0	0.067

Supplementary Table 2. Multiple linear regression studying the association between Appendicular Lean Mass Index, ALMI, (outcome variable) and age, time of follow-up, TWL% and nadir weight (predictor variables) in the RYGB cohort. TWL%, total weight loss.
